# Neuroprotective Effect of Mas Activation by BIO101 in Vincristine‐Induced Small Fiber Neuropathy

**DOI:** 10.1111/jns.70055

**Published:** 2025-08-20

**Authors:** Simon Frachet, Aurore Danigo, Mathilde Latil, Pierre J. Dilda, Flavien Bessaguet, Laurence Richard, Franck Sturtz, Laurent Magy, Claire Demiot

**Affiliations:** ^1^ NeurIT Neuropathies et Innovations Thérapeutiques UR 20218, Faculties of Medicine and Pharmacy University of Limoges Limoges France; ^2^ Department of Neurology, Reference Center for Rare Peripheral Neuropathies University Hospital of Limoges Limoges France; ^3^ Biophytis, Sorbonne Université – BC9 Paris France; ^4^ UMR INSERM 1083 CNRS 6015 MITOVASC Laboratory, CarMe Team University of Angers Angers France; ^5^ Transversal and Territorial Therapeutic Education Unit (UTTEP87) University Hospital of Limoges Limoges France

**Keywords:** 20‐hydroxyecdysone, chemotherapy‐induced peripheral neuropathy, drug repositioning, neuropathic pain, neuroprotection

## Abstract

**Background and Aims:**

Chemotherapy‐induced peripheral neuropathy (CIPN) is a significant side effect that limits the dosage of many anticancer therapies, such as vincristine. At present, there are no effective pharmacological treatments to prevent CIPN. The Mas receptor (MasR) is expressed in the peripheral nervous system and plays a role in pain modulation. While the antinociceptive properties of MasR activation in CIPN have been documented, its potential neuroprotective effects have not been explored in the peripheral nervous system. BIO101, a highly purified form of the MasR activator 20‐hydroxyecdysone, exhibits a positive safety profile in a Phase 1 study without any serious adverse events.

**Methods:**

This study aimed to investigate the neuroprotective effects of BIO101 in a mouse model of vincristine‐induced peripheral neuropathy (VIPN). Swiss mice were treated with daily doses of vincristine. VIPN was evaluated through repeated measurements of tactile sensitivity, quantification of intraepidermal nerve fibers (IENF) and dorsal root ganglion (DRG) neurons, and ultrastructural analysis of the sciatic nerve.

**Results:**

Vincristine led to mechanical allodynia and reduced the density of IENF, DRG neurons, and unmyelinated nerve fibers in the sciatic nerve. Prophylactic administration of BIO101 mitigated vincristine‐induced symptoms and nerve damage. The neuroprotective effect of BIO101 was nullified when the MasR antagonist A779 was administered; confirming the involvement of MasR.

**Interpretation:**

Therefore, BIO101 emerges as a safe and promising preventive treatment against vincristine‐induced small fiber neuropathy.

## 1 Introduction

Chemotherapy‐induced peripheral neuropathy (CIPN) represents a significant dose‐limiting adverse effect of various anticancer agents [1]. Vincristine is notably associated with peripheral neurotoxicity in malignant hematologic conditions. Vincristine‐induced peripheral neuropathy (VIPN) typically presents as sensory loss and neuropathic pain. This significantly affects the quality of life of individuals during and sometimes long after cancer therapy. The primary strategy for addressing VIPN involves reducing or discontinuing treatment regimens containing vincristine. However, this approach may compromise patient survival, emphasizing the necessity for the development of neuroprotective medications.

The renin–angiotensin system (RAS), extensively studied for its role in regulating arterial blood pressure, is also widely expressed in the peripheral nervous system [2]. Previous studies have demonstrated that modulation of the RAS by angiotensin‐converting enzyme inhibitors (ACEI) or angiotensin receptor blockers (ARB) is neuroprotective in murine models against peripheral neuropathy induced by paclitaxel [3], oxaliplatin [4], and vincristine [5]. However, analyzing a prospective cohort of patients undergoing a neurotoxic regimen revealed weak neuroprotective effects of ACEI and ARB [6]. This indicates the necessity for a novel pharmacological approach to modulate the RAS.

The Mas receptor (MasR) is a G‐protein‐coupled receptor that binds angiotensin‐(1‐7) (Ang‐1‐7), a peptide in the RAS cascade [7]. MasR is expressed in the dorsal root ganglia (DRG) [8, 9] and sciatic nerves of mice. During the acute phase of an axotomy, MasR is overexpressed in the sciatic nerve of the mouse; then its expression decreases several days later at the same time as functional recovery, suggesting a role of MasR during nerve healing [10]. Treatment with the MasR antagonist, A779, impairs functional recovery in a mouse model of sciatic nerve crush injury [11]. Administration of angiotensin‐(1‐7) mimics exercise‐induced analgesia in a rat model of chronic sciatic nerve constriction [12]. Activation of MasR would therefore have anti‐nociceptive and neuroregenerative properties in the peripheral nervous system. BIO101 is a drug candidate developed by Biophytis for treating sarcopenia [13] and COVID‐19 [14]. The active ingredient in BIO101 is a highly purified form of 20‐hydroxyecdysone, a polyhydroxylated phytosteroid that activates MasR [15]. Thus, the aim of this study was to investigate if BIO101, by activating MasR, demonstrates antinociceptive and neuroprotective effects in a mouse model of VIPN.

## 2 Methods and Materials

This study was approved by the French Ministry of Higher Education and Research (APAFIS#40571‐2023013016173443v2) and adhered to the European Community Guidelines for the Ethical Care of Experimental Animals (Directive 2010/63/EU). The experiments were conducted in accordance with the ARRIVE guidelines [16]. Every attempt was made to reduce suffering and minimize the animal count in subsequent experiments.

### 2.1 Animals and Treatments

Fifty adult male and female Swiss mice (4–5 weeks old, sex ratio = 1:1) were obtained from Janvier labs (Saint Berthevin, France). The mice were housed in plastic cages and subjected to a 12 h light/dark cycle with ad libitum access to food and water. The shredded paper was provided as nesting material for environmental enrichment (BISCEm‐animal care and facility center).

As previously described [5], VIPN was induced by eight (morning) intraperitoneal (i.p.) injections of vincristine (100 μg/kg/day, vincristine Hospira, Pfizer, New York, NY, USA). An equivalent volume of vincristine vehicle was administered to control (Ctrl) mice (NaCl 0.9%, i.p.). Although the intravenous route theoretically best mimics human administration, the i.p. route was technically preferred due to its simplicity, reproducibility, lower risk of venous damage and chemotherapy leakage, and reduced stress for the animals—an important factor in models assessing pain sensitivity. It is generally acknowledged that i.p. administration is appropriate in preclinical models when the aim is to assess target engagement rather than detailed pharmacokinetics [17].

Treatment with BIO101 (25 mg/kg/day, i.p.; Biophytis, Paris, France) or with its vehicle (Veh; equivalent volume of NaCl 0.9%, i.p.) began 1 day (evening) ahead of the first vincristine administration and was sustained daily for the duration of the protocol. The dosage of BIO101 was proposed by Biophytis based on unpublished pharmacokinetic data. The specific antagonist of MasR, A779 (0.20 μg/kg/day, MedChemExpress, New Jersey, USA) was administered subcutaneously at the same time as BIO101 or its vehicle.

The mice were randomly assigned to five groups as follows: (1) VEH‐Ctrl, which received only NaCl 0.9%; (2) VEH‐VCR, which received NaCl 0.9% and vincristine; (3) BIO101‐Ctrl, which received BIO101 and NaCl 0.9%; (4) BIO101‐VCR, which received BIO101 and vincristine; and (5) A779+BIO101‐VCR, which received A779, BIO101, and vincristine. Mice were assigned to each group using an online randomization tool (http://www.graphpad.com/quickcalcs/index.cfm, accessed on April 24, 2023). The number of 10 animals per group was determined based on the von Frey test results (mean and standard deviation) obtained during a study on the VIPN model [5], and confirmed using the G Power tool.

Tactile sensitivity was evaluated through the von Frey filament test. At the end of experiments (D8), paw skin, L4‐L5 dorsal root ganglia (DRG), and sciatic nerve were promptly excised for immunofluorescence and ultrastructural analyses [5]. Nervous tissues were removed from six animals per group. In previous studies, a significant difference could be observed between control and vincristine‐treated groups only with *n* = 6. These six animals were randomly designed. From the randomization until histological analyses, treatment groups were coded to maintain blinding. Thus, behavioral assessments and counting experiments were conducted by assessors who were unaware of the treatment assignments (Veh, BIO101 or the combination of A779+BIO101‐VCR) and experimental conditions (Ctrl or VCR).

### 2.2 Mechanical Allodynia

Von Frey filaments (Bioseb, Vitrolles, France) were utilized to assess tactile sensitivity in mice [18]. The mice were housed in a plastic cage with a wire mesh floor, allowing access for their paw examination. To minimize visual stimuli, the cage was shielded with an opaque cup. The tactile sensitivity of the mid‐plantar left hind paw was evaluated using an up‐down method to establish the mechanical threshold [19]. Each animal underwent three testing sessions for each experimental condition.

**FIGURE 1:**
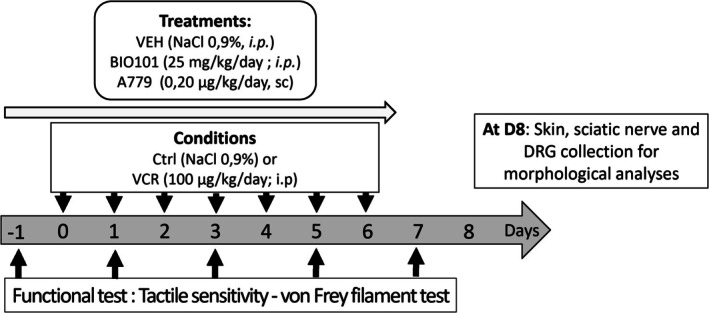
Schematic of the study design. Ctrl, control; DRG, dorsal root ganglion; PTX, paclitaxel; VCR, vincristine; VEH, vehicle.

The experiments were conducted on the reference day (RD) to assess the baseline for each animal, on Day 1 (D1) corresponding to the second vincristine injection, and on D3 and D7 after the initial vincristine injection (Figure 1).

### 2.3 Immunofluorescence of Footpad Skin

Intraepidermal nerve fiber (IENF) density was determined by quantifying nerve fibers observed through protein gene product 9.5 (PGP9.5) staining of the footpad skin, following the procedure outlined in a previous study [20]. Footpads were excised using a 3 mm punch biopsy, fixed in 4% paraformaldehyde (PFA) overnight, cryoprotected in 30% sucrose, and stored at −20°C. Tissue sections of 20 μm thickness were prepared using a cryostat and then subjected to overnight incubation with a primary antibody targeting PGP9.5 (rabbit monoclonal, 1:50 dilution; Abcam, Cambridge, UK), followed by treatment with a Cy3‐conjugated secondary antibody (1:500 dilution; Jackson ImmunoResearch, Suffolk, UK). Epidermal nerve fibers were quantified under blinded conditions at 400× magnification (Eclipse 50i, Nikon Europe B.V., Amstelveen, The Netherlands), in accordance with the guidelines of the European Federation of Neurological Societies [21]. Epidermal nerve density was calculated as the ratio of epidermal nerves to the length of the dermo‐epidermal junction (IENF number/mm). Three slides per mouse were examined.

### 2.4 Morphological Analysis of Sciatic Nerves

Sciatic nerves were processed for electron microscopy as previously described [5]. In summary, the nerves were dissected, placed in a 2.5% glutaraldehyde solution diluted in Sorensen buffer, dehydrated, and embedded in Epon 812 resin (Euromedex, France). Semi‐thin sections were stained with toluidine blue, while ultrathin sections were stained with uranyl acetate and lead citrate. Using an electron microscope (JEM‐1400 Flash, Jeol, Peabody, MA, USA), four photographs per animal were captured at a magnification of 3000×. The count of myelinated and unmyelinated nerve fibers per millimeter [2] was conducted to determine the density.

### 2.5 Data Analysis

All data are presented as the mean ± SEM. A nonparametric Kruskal–Wallis test and Dunn's multiple comparisons test were utilized for data that did not adhere to a Gaussian distribution. Statistical significance was defined as *p* < 0.05.

## 3 Results

### 3.1 Restoration of Normal Tactile Sensitivity by BIO101 at 25 mg/kg

**FIGURE 2:**
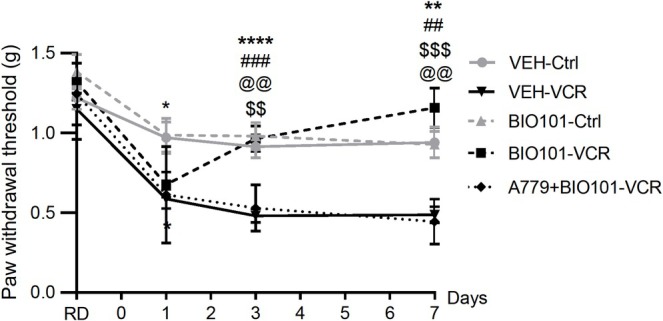
Effects of BIO101 on mechanical allodynia induced by vincristine in mice. Mechanical sensitivity was evaluated using the von Frey filament test. *n* = 6–8 mice per group, *p < 0.05, **p < 0.01, ****p < 0.0001, VEH‐VCR versus VEH‐Ctrl. ##*p* < 0.01, ###*p* < 0.001 VEH‐VCR versus BIO101‐VCR. @@*p* < 0.05, @@@*p* < 0.001 A779+BIO101‐VCR versus BIO101‐VCR. $$*p* < 0.01 A779+BIO101‐VCR versus VEH‐Ctrl. Ctrl, control; VCR, vincristine; VEH, vehicle.

Animals receiving vincristine, BIO101, or a combination of the two showed no significant difference in weight gain compared with control animals, suggesting good tolerability of the drugs in mice (Figure S1). Mice exposed to vincristine developed mechanical allodynia from D1 to D7. A significant difference in the paw withdrawal threshold was observed between mice in the VEH‐VCR group and those in the VEH‐Ctrl group at D1 (*p* = 0.0136), D3 (*p* < 0.0001), and D7 (*p* = 0.002). In mice exposed to vincristine, BIO101 induced a complete recovery of normal mechanical sensitivity from D3 (*p* = 0.0004) to D7 (*p* = 0.0012, BIO101‐VCR vs. VEH‐VCR). There was no significant difference between the BIO101‐VCR and the VEH‐Ctrl groups at D1 (*p* = 0.1211), D3 (*p* = 0.9650), and D5 (*p* = 0.5285). In combination with A779, a specific MasR antagonist, the beneficial effect of BIO101 on vincristine‐induced allodynia was abolished (D3: *p* = 0.0029, D7: *p* = 0.0008, BIO101‐VCR vs. A779+BIO101‐VCR). Mice treated with A779+BIO101 exhibited tactile sensitivity similar to that of the VEH‐VCR mice, that is, tactile allodynia from D3 (*p* = 0.0029) to D7 (*p* = 0.0014, A779+BIO101‐VCR vs. VEH‐Ctrl, Figure 2).

### 3.2 Vincristine‐Induced Loss of Sensory Nerve Endings and Neurons Prevented by BIO101 at 25 mg/kg

**FIGURE 3:**
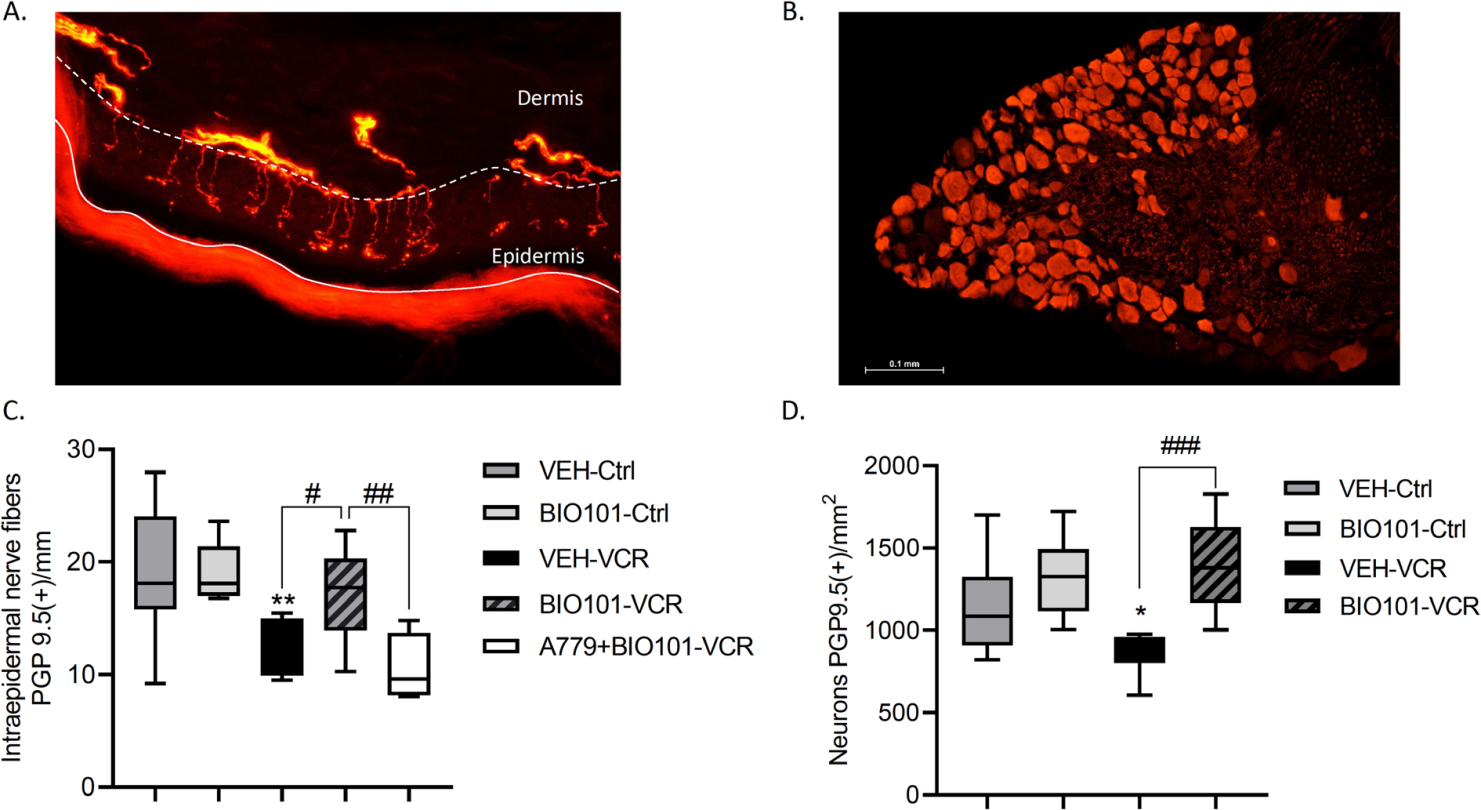
Effect of BIO101 at 25 mg/kg on the loss of sensory nerve endings and sensory neurons induced by vincristine in mice. Immunohistochemistry for PGP9.5 was performed on (A) paw skin and (B) DRG sections. (C) Intraepidermal nerve fiber density. Three sections of paw skin were examined per mouse. *n* = 6 mice. (D) Quantification of DRG neuron density. Three DRG sections and three DRG per mice were counted. *n* = 6 per group. *p < 0.05, **p < 0.01 VEH‐VCR versus VEH‐Ctrl. #*p* < 0.05, ###*p* < 0.001 BIO101‐VCR versus VEH‐VCR. Ctrl, control; PGP9.5, protein gene product 9.5; VCR, vincristine; VEH, vehicle.

BIO101 at 25 mg/kg did not affect IENF and DRG neuron densities in the control group. The IENF density was notably lower in VEH‐VCR mice compared to VEH‐Ctrl mice (*p* = 0.0091). BIO101 treatment partially mitigated this decrease in IENF density (*p* = 0.0311, BIO101‐VCR vs. VEH‐VCR, Figure 3A,B). Moreover, there was no significant difference in IENF density between the VEH‐Ctrl group and the BIO101‐VCR group (*p* = 0.7862). In combination with A779, the beneficial effect of BIO101 on vincristine‐induced decrease in IENF was abolished (*p* = 0.0444, BIO101‐VCR vs. A779+BIO101‐VCR and *p* = 0.88, VEH‐VCR vs. A779+BIO101‐VCR).

Similarly, DRG neuron density significantly decreased after vincristine administration (*p* = 0.0462; VEH‐Ctrl vs. VEH‐VCR). Pre‐treatment with BIO101 prevented the vincristine‐induced decline in DRG neurons (*p* = 0.0003, BIO101‐VCR vs. VEH‐VCR, Figure 3C,D). There was no significant difference in DRG neuron density between the VEH‐Ctrl group and the BIO101‐VCR group (*p* = 0.3067).

### 3.3 Vincristine‐Induced Loss of Unmyelinated Axons in the Sciatic Nerve Prevented by BIO101 at 25 mg/kg

**FIGURE 4:**
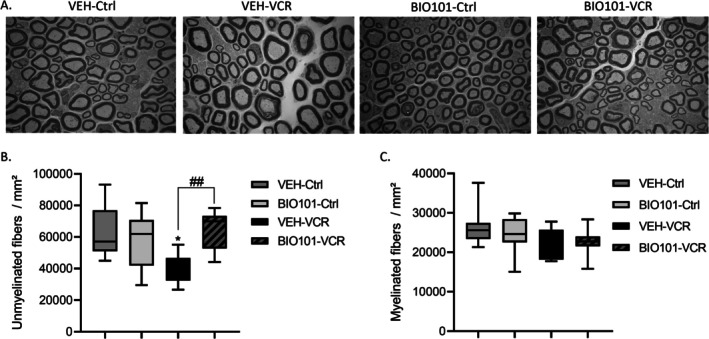
Quantitative analysis of sciatic nerves through electron microscopy. (A) Visualization of myelinated fibers in the sciatic nerve. (B) Quantification of unmyelinated nerve fiber density. (C) Quantification of myelinated nerve fiber density. *n* = 6 per group; **p < 0.01 VEH‐VCR versus VEH‐Ctrl, ##*p* < 0.01; BIO101‐VCR versus VEH‐VCR. Ctrl, control; VCR, vincristine; VEH, vehicle.

BIO101 at 25 mg/kg did not impact the morphology and quantity of unmyelinated and myelinated nerve fibers in the sciatic nerve of the Ctrl group (Figure 4A). The density of unmyelinated nerve fibers in the sciatic nerve was notably lower in the VEH‐VCR mice compared to the VEH‐Ctrl mice (*p* = 0.0113). Administration of BIO101 from D‐1 prevented this reduction in unmyelinated nerve fiber density (*p* = 0.0032, BIO101‐VCR vs. VEH‐VCR and *p* = 0.9539 VEH‐Ctrl vs. VCR‐BIO101, Figure 4B).

There was no significant disparity in myelinated fiber density between VEH‐VCR and VEH‐Ctrl mice (Figure 4C).

## 4 Discussion

The primary finding of this study was that BIO101 restored normal tactile sensitivity in a well‐established murine model of VIPN. Additionally, it alleviated the reduction in densities of IENF, unmyelinated nerve fibers in the sciatic nerve, and DRG neurons, indicating a neuroprotective effect. These beneficial effects were reversed when A779, a selective MasR antagonist, was co‐administered; suggesting that the neuroprotective effect of BIO101 is probably facilitated through MasR.

Under our experimental conditions, vincristine induced a sensory small fiber neuropathy characterized by pronounced mechanical allodynia, along with impairment of small nerve fiber endings and axons, consistent with the early stages of VIPN in humans. This condition typically leads to chemotherapy cessation before irreversible large fiber involvement and, in some cases, long‐term neuropathic pain. However, VIPN is not exclusively a small fiber neuropathy. Large fibers, which were not specifically assessed behaviorally or neurophysiologically in this experimental context, were not affected at the axonal level, as determined by sciatic nerve biopsies. To enhance the translational relevance of the observed neuroprotective effect of BIO101, future research may need to explore higher dosing and extended exposure durations to also assess large fiber protection in VIPN. BIO101 was administered just prior to chemotherapy to prevent vincristine‐induced neuropathic pain and sensory nerve fiber injury. This preventive measure is particularly relevant for CIPN, as chemotherapy treatments are typically planned and administered in a hospital setting. According to ASCO and ESMO guidelines, there is currently no recommended strategy for preventing CIPN in patients receiving chemotherapy [22, 23]. Duloxetine is the sole drug recommended for managing CIPN, specifically for pain relief in affected patients. Drug repositioning strategies are significant for CIPN prevention due to the failures of many drug candidates in clinical trials.

In our experimental condition, the co‐administration of the MasR antagonist A779 and BIO101 counteracted the beneficial effect of BIO101 on the mechanical allodynia induced by vincristine. This result confirmed that the antinociceptive effect of BIO101 was mediated by MasR. Moreover, neuropathological analyses showed that the protective effect of BIO101 on the loss of IENF induced by vincristine was also inhibited by the co‐treatment with A779. Thus, BIO101 exerted a neuroprotective effect on the sensory small nerve fibers affected by VCR, an effect mediated through the MasR. Previous studies have reported that stimulating MasR produces an antinociceptive effect in various pain models [8, 24, 25, 26]. Our results demonstrate that activating MasR with BIO101 not only serves as a symptomatic treatment but also provides neuroprotective benefits by preventing vincristine‐induced loss of the sensory nerve fibers (decrease of IENF and of unmyelinated nerve fiber density in the sciatic nerve) and sensory neurons. Several studies have highlighted the neuroprotective role of the Ang1‐7/Mas axis in the central nervous system [27], presumably through an antioxidant mechanism [28]. However, as of now, no scientific study has translated this effect into CIPN.

The neuroprotective effects of RAS inhibitors (ACEI or ARB) on the peripheral nervous system, observed in mouse models of peripheral neuropathy, are believed to be mediated by a shift in the RAS cascade from AT1R to angiotensin II type 2 receptor (AT2R) [29]. The latter promotes neurite outgrowth and restores Akt signaling [30]. More recently, MasR, bound to by Ang 1‐7, a biologically active peptide resulting from the degradation of angiotensin I or angiotensin II, was discovered [31]. This receptor, similar to AT2R, is also thought to counterbalance the pro‐fibrotic and pro‐inflammatory effects of AT1R. Although the involvement of MasR in neuropathic pain through the inhibition of spinal c‐fos expression [32] and calcium channels in the DRGs [33] has been investigated, its potential neuroprotective effects in the context of VIPN remain unexplored. Additionally, previous preclinical studies on MasR have mostly used its natural agonist Ang 1‐7, which has a half‐life of 29 min in humans [34] and is cleaved into several peptides that do not activate MasR. Since BIO101 is not peptidic and has a longer half‐life (2.4–4.9 h) [35], a stronger MasR‐related neuroprotective effect is expected.

BIO101 is currently in the clinical stage for various reasons. Apart from preclinical models, BIO101 showed a favorable safety profile in a Phase 1 study, with no serious adverse events reported in healthy young and older adult volunteers [35]. A recently published Phase 3 study revealed a significant decrease in the risk of death and respiratory failure due to COVID‐19 [14]. Additionally, BIO101, which exhibited promising results in animal models [36] underwent a double‐blind, randomized interventional Phase 2b clinical trial in older adults with sarcopenia at risk of mobility disability [13]. Since sarcopenia is linked to adverse health outcomes, particularly in cancer patients [37], a treatment addressing both sarcopenia and providing neuroprotection against CIPN could substantially enhance the quality of life of these patients.

A significant consideration in administering neuroprotective agents to cancer patients is the risk of interfering with chemotherapy and inadvertently stimulating cancer cell proliferation. However, there is a need for in vitro investigations on 20‐hydroxyecdysone, the active ingredient in BIO101. Previous studies have shown the suppression of cancer cell proliferation [38, 39, 40] and a chemo‐sensitizing effect [41, 42], indicating the possible safe application of BIO101 in cancer treatment without compromising its efficacy.

Therefore, BIO101 showed a favorable safety profile in a Phase 1 study, with no serious adverse events reported in healthy young and older adult volunteers. It would be interesting to replicate these results using other CIPN models.

## Conflicts of Interest

Biophytis provided University of Limoges with BIO101 for the whole duration of the study. Mathilde Latil and Pierre J. Dilda are employees of Biophytis and own stocks in the company.

## Supporting information


**Figure S1.** Effects of BIO101, vincristine and A779 on weight gain. Abbreviations: Ctrl, control; VCR, vincristine; VEH, vehicle.

## Data Availability

Data sharing not applicable to this article as no datasets were generated or analysed during the current study.
