# Supplementary figures and images for: Neuroprotective Effect of Mas Activation by BIO101 in Vincristine‐Induced Small Fiber Neuropathy
Source: J Peripher Nerv Syst. 2025 Aug 20;30(3):e70055. doi: 10.1111/jns.70055 (PMC12366684; doi:10.1111/jns.70055)

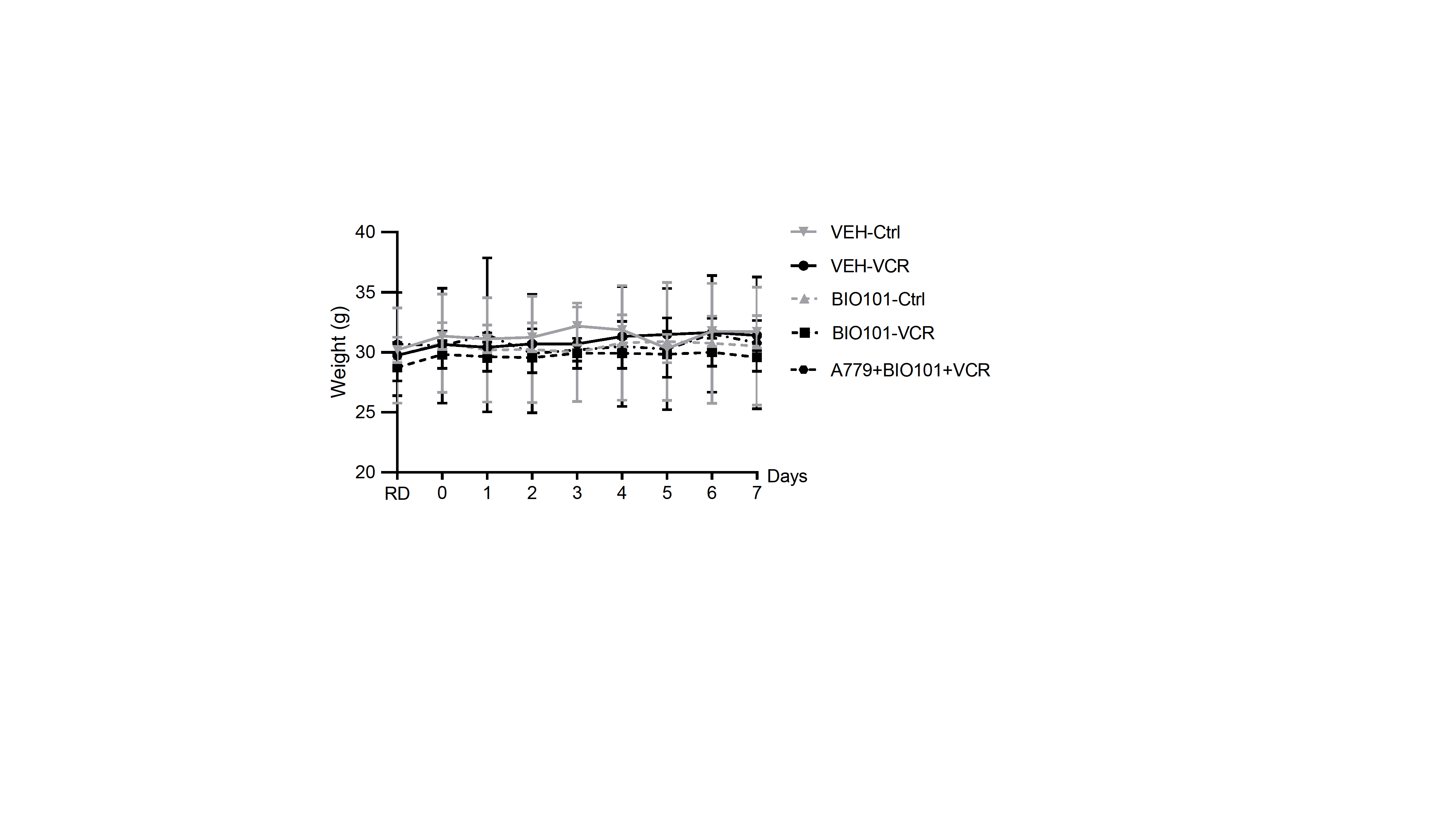

Supplement: Supplementary file 1 — Figure S1. Effects of BIO101, vincristine and A779 on weight gain. Abbreviations: Ctrl, control; VCR, vincristine; VEH, vehicle. [file JNS-30-0-s001.tif]
